# Modeling and dynamic analysis of tuberculosis in mainland China from 1998 to 2017: the effect of DOTS strategy and further control

**DOI:** 10.1186/s12976-020-00124-9

**Published:** 2020-05-04

**Authors:** Siyu Liu, Yingjie Bi, Yawen Liu

**Affiliations:** 1 School of Public Health, Jilin University, Xinmin Street 1163, Changchun, 130021 China; 2 School of Mathematics, Jilin University, Qianjin Street 2699, Changchun, 130012 China

**Keywords:** Epidemic model, Tuberculosis, DOTS strategy, Optimal control

## Abstract

**Background:**

Tuberculosis (TB) is one of the most important health topics in the world. Directly observed treatment and short course chemotherapy (DOTS) strategy combines medicine care and modern health system firmly, and it has been carried out by World Health Organization (WHO) since 1997. In the struggle with TB, China has promoted the process of controlling the disease actively, and the full coverage of DOTS strategy has been reached around 2004. Mathematical modeling is a very useful tool to study the transmission of diseases. Understanding the impact of DOTS strategy on the control of TB is important for designing further prevention strategy.

**Methods:**

We investigate the impact of control strategy on the transmission of TB in China by dynamic model. Then we discuss further control for TB aiming at developing new vaccine and improving treatment. The optimal control problem, minimizing the total number of infectious individuals with the lowest cost, is proposed and analyzed by Pontryagin’s maximum principle. Numerical simulations are provided to illustrate the theoretical results.

**Results:**

Theoretical analysis for the epidemic model is given. Based on the data reported by National Bureau of Statistics of China (NBSC), the basic reproduction number of each stage is estimated and compared, and they are $\mathcal {R}_{0}^{1}=1.7885$ and $\mathcal {R}_{0}^{2}=1.0741$, respectively. Optimal control strategy for further control is designed and proved well. An intuitionistic comparison between the optimal control strategy and the current control strategy is given.

**Conclusions:**

The diagnosis and treatment of TB in China have been promoted a lot and the $\mathcal {R}_{0}$ is reduced by the full coverage of DOTS strategy. However, the $\mathcal {R}_{0}$ in China is still greater than 1 now. The relationship between $\mathcal {R}_{0}$ and vaccination strategy is shown. Optimal strategy aiming at exposed and infected population is suggested for further control.

## Background

Tuberculosis caused by Mycobacterium tuberculosis (Mtb) is a widespread and deadly chronic infectious disease. Generally speaking, the Mtb usually affects the lungs. The disease is transmitted through droplets from the throat and lungs of people with acute infection, and about one-third of the world’s population has been infected with Mtb nowadays [1]. The Mtb carriers may become infectious individuals all their lives. The probability of changing from the latent state to active state is only about 1/10 to 1/5. However, in the statistics of 2017 [2], about 10.0 million of the people get TB, of which 59% are new cases. TB is still one of the top 10 causes of death. The prevention, diagnosis and treatment of TB need adequate funding. Because TB is a chronic disease, the material support must be sustained for a long time. In the recent decade, the finance support for the prevention of TB has increased, but funding gaps still exist. The eradication of TB is a worldwide scale challenge especially in South-East Asia and Africa. Chemotherapy and antibiotics have become powerful measures for the elimination of disease since 20th century. Most deaths of TB can be prevented with early diagnosis and effective treatment which had saved about 54 million of people between 2000 and 2017 [2]. DOTS strategy is a control measure formulated and carried out by WHO to end the transmission of TB. A lower proportion of recent transmissions is observed in the county with long-term DOTS implementation [3] and DOTS strategy is now considered as the most suitable control measure for the cost-effective principle. China is one of the highest TB burden countries in the world, more than 80% infectious are in the mid-western regions of China and the drug-resistance problem is very serious. For the prevention and control of TB, Chinese government has taken a series of measures including the universal coverage of DOTS strategy. The implement of this strategy has made prominent improvement on diagnosis and treatment for TB.

In the past several decades, mathematical model is playing an important role in disease control. The dynamic system can describe the transmission process of TB. Differential equation models are widely used in the study of TB, particularly, complex network models are applied to describe the complexity because of topological characterization. Five different complex network models are established to study the transmission of TB and to help to eradicate the disease [4]. Data fitting gives a proper combination of theoretical analysis and practical situation. Demographic and epidemiological data have been used to explore the effects of population growth, randomness, clustering of contacts and age structure on TB dynamics in [5]. Now there are several works focused on TB, which have been studied through many factors, such as fast and slow progression [6], drug-resistant strains [7], reinfection [8, 9], co-infection [10], migration and seasonality [11, 12]. For the prevention of TB, the model formulated with isolation [13], treatment [14], vaccination [15] or a combination of different control strategies [16–18] are discussed. On the other hand, if the people take initiative on the awareness of prevention and control, the effect will be better. Das et al. [19] investigate the influence of social awareness spread by media in TB transmission dynamics and the corresponding optimal control strategy with minimum cost is also given. To make a better control, optimal control theory is applied in many areas, such as the design of therapy [20], optimal control of the disease among animals [21] and best prevention strategy for TB [22, 23]. Nowadays, the impact of DOTS strategy on the control of tuberculosis in China is scarce, moreover, the study of the whole situation of tuberculosis in China is still absent since the reported data is available.

The new reported cases of tuberculosis in China increases sharply around the year of 2004, so the study is divided into two stages. In this paper, we study the TB trends mainly focused on the full coverage of DOTS strategy in China in recent two decades. Theoretical analysis for the dynamic system and the estimation of the basic reproduction number for before and after the 100% coverage of DOTS strategy which is based on the reported TB cases in China are given. We study the efforts of DOTS strategy through the basic reproduction number of the two stages. The full coverage of DOTS strategy has reduced the level of TB obviously, but the disease still exists ($\mathcal {R}_{0}>1$). Then various further controls for TB are analyzed and simulated. Our theoretical results and numerical simulations do great help to design optimal control strategy in practice.

## Methods

### Mathematical model

In this section, we formulate a dynamic model to investigate the current situation of TB transmission in China. In this model, the total size of population at time *t* is divided into five subclasses: susceptible, vaccinated, exposed, infectious and recovered denoted by *S*,*V*,*E*,*I* and *R*, respectively. Because the vaccination strategy for TB in China focuses on newborns, the subclass *V* is regarded as the newborns vaccinated successfully. During the protection period of BCG, people cannot get infected, even if they contact with infectious individuals. But the time-efficiency of BCG is very limited, the vaccinated individuals may lose immunity and become susceptible. The susceptible class is increased at recruitment rate *Λ* and at the rate of *k* which is from vaccinated class by losing temporary immunity. The parameter *p*∈[0,1] indicates the fraction of the newborns vaccinated successfully. *G*(*β*) is the transmission coefficient. In the first stage, i.e., the time from 1998 to 2003, we take *G*(*β*)=*β*; in the second stage, i.e., the year from 2004 to 2017, *G*(*β*) is taken as *ωβ* in which *ω* measures the increasing of the reported rate due to the improvement of diagnosis. Bilinear incidence *G*(*β*)*SI* is used to represent the rate at which the susceptible individuals are infected. *d* is the natural death rate coefficient, *σ* is the disease-induced death rate, *ε* is the rate of progression to active TB, and *γ*_1_ and *γ*_2_ denote the treatment rate for latent class and infectious class, respectively.

The above model description can be constructed as follows:

1 $$ \left\{\begin{aligned} \frac{{dS}}{{dt}}&=\Lambda(1-p)-G(\beta) SI+kV-dS, \\ \frac{{dV}}{{dt}}&=\Lambda p-kV-dV, \\ \frac{{dE}}{{dt}}&=G(\beta) SI-\gamma_{1}E-\varepsilon E-dE, \\ \frac{{dI}}{{dt}}&=\varepsilon E-dI-\sigma I-\gamma_{2}I, \\ \frac{{dR}}{{dt}}&=\gamma_{1}E+\gamma_{2}I-dR. \end{aligned} \right.  $$

Let *N* denote the total population size

2 $$ N(t)=S(t)+V(t)+E(t)+I(t)+R(t).  $$

Then, it follows from (1) that

3 $$ \frac{{dN}}{{dt}}=\Lambda-d(S+V+E+I+R)-\sigma I\leq \Lambda-dN.  $$

As *t*→*∞* in (3), the total population size *N*→*Λ*/*d*. It implies that the positively invariant set for system (1) is

4 $$ \Omega=\{(S, V, E, I,R)\in \mathbb{R}^{5}_{+}: 0\leq S+V+E+I+R\leq \Lambda/d\}.  $$

The dynamics behaviors of system (1) on the set *Ω* is sufficient to study in the following parts.

## Results

### Dynamics analysis

There is a unique infection-free equilibrium of system (1):

$$ E_{0}=\left(\frac{{\Lambda(k+d-pd)}}{{d(k+d)}}, \frac{{\Lambda p}}{{k+d}}, 0, 0, 0\right).  $$

Then by the principle of the next generation matrix in [24], we can obtain the basic reproduction number

5 $$ {}\mathcal{R}_{0}=\rho\left(FV^{{-1}}\right)=\frac{{G(\beta)\varepsilon\Lambda(k+d-pd)}}{{d(k+d)(d+\sigma+\gamma_{2})(\varepsilon+d+\gamma_{1})}},  $$

where *ρ* represents the spectral radius of matrix *FV*^−1^ and the matrices *F* and *V* are given by

$$ {\begin{aligned} F=\left(\begin{array}{cc} 0&\frac{{G(\beta)\Lambda(k+d-pd)}}{{d(k+d)}}\\ 0&0 \end{array} \right), \hspace{2em} V=\left(\begin{array}{cc} \varepsilon+d+\gamma_{1}&0\\ -\varepsilon&d+\sigma+\gamma_{2} \end{array} \right). \end{aligned}}  $$

*ε*/(*ε*+*d*+*γ*_1_) is the fraction of latent population becoming infected among those exiting the *E* class and 1/(*d*+*δ*+*γ*_2_) represents the mean duration of exiting the *I* class. We have the following results, and the relevant proof can be found in Appendix.

#### **Theorem 1**

The infection-free equilibrium *E*_0_ is locally asymptotically stable if $\mathcal {R}_{0}<1$ and unstable if $\mathcal {R}_{0}>1$.

#### **Theorem 2**

For system (*1*), the infection-free equilibrium *E*_0_ is globally asymptotically stable if $\mathcal {R}_{0}< 1$.

### Estimation and analysis of $\mathcal {R}_{0}$

By the above analysis, we know that the basic reproduction number is an important threshold for the control of disease. In order to estimate the value of basic reproduction number, we use the data of annual reported TB cases which have been released by the National Data [25] of China to do data fitting. In this paper, we study the reported data form 1998 to 2017. Notice that, during the period of 2004 to 2005, China has completed the 100% coverage of DOTS strategy for TB, and the diagnosis and treatment have been improved a lot. We divides the time into two stages (before and after the full coverage of DOTS strategy) to study the transmission and control of TB in China. The parameters are estimated with the reported data by least square method (LSM).

**Fig. 1:**
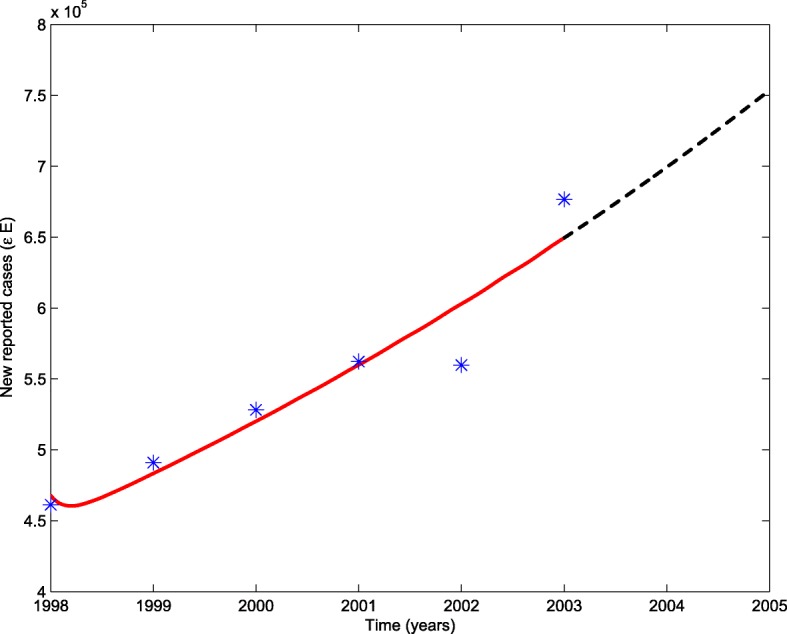
Goodness of fit for the first stage of TB trends

For the first stage, the time period is from 1998 to 2003 in which the coverage rate of DOTS in China is about 60%. By fitting system (1) to the annual reported TB cases, we obtain the estimations for the transmission rate *β* and the recovery rate of infectious *γ*_2_, which are listed in Table 1 and the goodness of fit is shown in Fig. 1, stars represent the newly reported TB cases by year, the red solid line shows the fit based on the true data and the dashed line shows the estimation of new reported cases in 2004 and 2005. In our estimate, the new reported cases in 2004 should be 7.0×10^5^, but actually the collected data in 2004 is 9.7×10^5^. It implies that the diagnosis of tuberculosis has been improved a lot. Using the estimated parameter values, we calculate the basic reproduction number $\mathcal {R}_{0}^{1}$ of the first stage is 1.7885.

**Table 1:** Parameter values

Parm	Description	Value	Source
*Λ*	Recruitment rate	1.6×10^7^*personyear*^−1^	[26, 27]
*d*	Natural death rate		
	first stage	0.0143 *year*^−1^	[26]
	second stage	0.0139 *year*^−1^	[27]
*G*(*β*)	Transmission rate		
	of infected population		
	first stage (*β*)	2.5338×10^−10^*person*^−1^*year*^−1^	LS
	second stage (*ωβ*)	5.0554×10^−10^*person*^−1^*year*^−1^	LS
*σ*	Disease-induced death rate	0.06 *year*^−1^	[12, 28]
*ε*	Rate of progression to infec-	6 *year*^−1^	[29]
	tious stage from the exposed		
*k*	Rate of waning immunity	0.25 *year*^−1^	[29–31]
*γ*_1_	Recovery rate of the exposed	0.6683 *year*^−1^	[29]
*γ*_2_	Recovery rate of the infectious		
	first stage	0.0634 *year*^−1^	LS
	second stage	0.3972 *year*^−1^	LS
*p*	The fraction of BCG	0.6	[29–31]
	vaccinated successfully		

Parm, Parameter; LS, least square

**Fig. 2:**
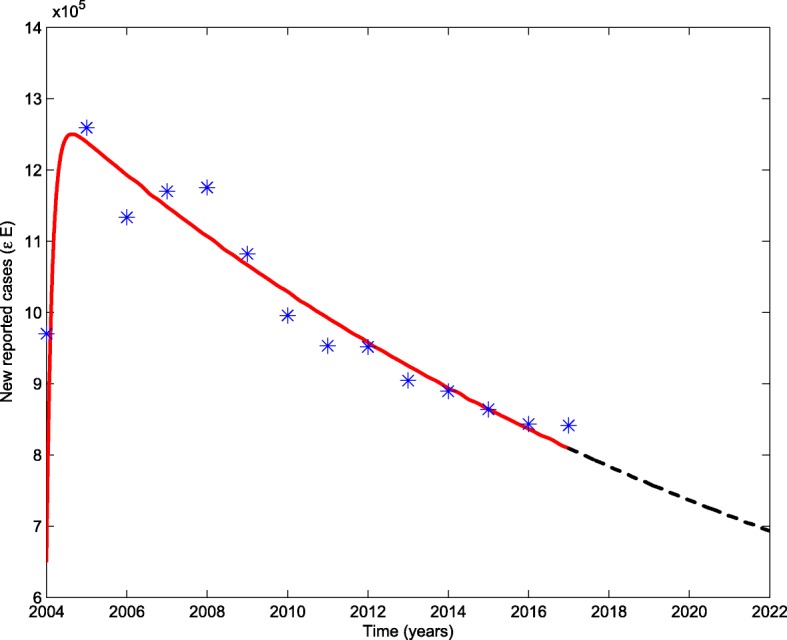
Goodness of fit for the second stage of TB trends

For the second stage, we fit system (1) to the annual reported TB cases from 2004 to 2017. The DOTS coverage rate increased rapidly to 95% in 2004 and achieved 100% coverage since 2005. Compared with the first stage, not only for the control of TB, the integral medical level of China has promoted a lot, one of the most obvious reactions is the average lifetime has risen to about 72 years. To make a better data fit, we take the natural death rate as 0.0139*year*^−1^ in the second stage. With LSM, the transmission rate and the recovery rate of infectious are estimated and listed in Table 1. The goodness of fit is shown in Fig. 2 with red solid line and the black dashed line shows the trend of new reported cases under current control strategy. According to the parameter values we estimated, the basic reproduction number of the second stage is $\mathcal {R}_{0}^{2}=1.0741$.

Judging from the data we collected, the newly reported TB cases increase at a relatively stable speed in the first stage, but proliferate fast in the prophase of the second stage and then decrease year by year. It is closely related to the full coverage of DOTS strategy. In the prophase of the 100% coverage of DOTS strategy, the diagnosis has developed rapidly, the data we have studied comes from the annual summary of the number of TB patients in different levels of hospitals. Due to the improvement of diagnosis and the strengthening of screening, the newly reported TB cases increase first after the full coverage of DOTS strategy. It is reflected in the estimation of parameter *G*(*β*) in our model. The parameter *G*(*β*) in the second stage is larger than in the first stage. And the corresponding treatment measures have been improved a lot, it makes the newly reported TB cases decreased since 2009. It can be seen from the significantly increase recovery rate of the infectious (*γ*_2_). A more intuitionistic expression is that the period in which the infected individuals need to recover (1/*γ*_2_) is shorten sharply.

Comparing the basic reproduction number of the two stages, we can clearly see that under the full coverage of DOTS strategy, the control of TB has improved a lot and it is strongly related to the promotion of diagnosis and treatment. The decrease of the basic reproduction number indicates that the level of transmission for TB is declining in population. Although the newly reported TB cases in China have fallen in recent years and it seems that the disease is gradually vanished, however, the basic reproduction number is still greater than 1, the elimination of TB cannot achieve in this situation. The estimation of $\mathcal {R}_{0}$ is very much in line with previous studies [32–34]. In order to eradicate the disease, we need to take a further control.

### Further control for TB

According to the above results, although the newly reported TB cases in China have decreased since 2009 and after the full coverage of DOTS strategy the basic reproduction number has reduced obviously, the basic reproduction number is still greater than 1. Further control for TB must focus on the development of new vaccine and new method for diagnosis and treatment. First, we consider that if the new vaccine comes into use mainly from two sides: increase the vaccination rate and protection period.

To explore the effect of successful vaccination (*p*) on the basic reproduction number $\mathcal {R}_{0}$, we calculate the derivative of $\mathcal {R}_{0}$ with respect to the parameter *p* as follows

6 $$ \frac{{\partial \mathcal{R}_{0}}}{{\partial p}}=-\frac{{G(\beta) \varepsilon \Lambda}}{{(k+d)(d+\sigma+\gamma_{2})(\varepsilon+d+\gamma_{1})}}.  $$

**Fig. 3:**
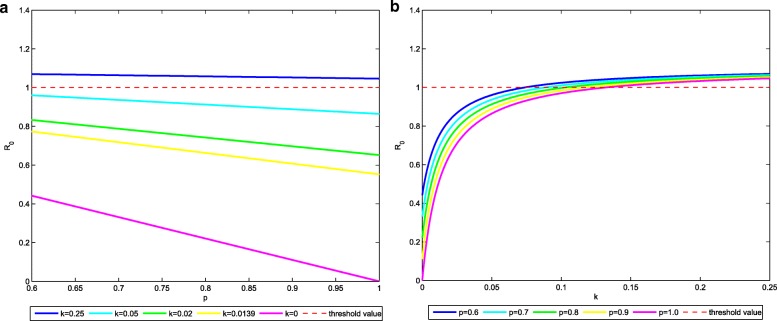
**a** The effect of *p* on $\mathcal {R}_{0}$ when *k* is varied. **b** The effect of *k* on $\mathcal {R}_{0}$ when *p* is varied

Because all the parameters are non-negative, $\partial \mathcal {R}_{0}/\partial p<0$. Figure 3a illustrates how the basic reproduction number depends on the various weights of *p* with different *k*: as *p* increases, the basic reproduction number decreases. All the other parameters are shown in Table 1. Therefore, increasing the fraction of successful vaccinated infants helps to reduce TB.

Similarly, to investigate the effect of extending the protection period of vaccination (1/*k*) on the basic reproduction number $\mathcal {R}_{0}$, direct calculation shows that

7 $$ \frac{{\partial \mathcal{R}_{0}}}{{\partial k}}=\frac{{G(\beta)\varepsilon\Lambda p}}{{(k+d)^{2}(d+\sigma+\gamma_{2})(\varepsilon+d+\gamma_{1})}}.  $$

Figure 3b shows how the basic reproduction number $\mathcal {R}_{0}$ depends on the various weights of *k* with different *p*: as *k* decreases, the basic reproduction number decreases. Since $\partial \mathcal {R}_{0}/\partial k>0$, increasing the protection period (1/*k*) can do active effect on TB control. All the other parameters are shown in Table 1.

Now, we consider how to give an optimal control for TB through the development of treatment. We mainly discuss on the estimation of parameter *G*(*β*) and *γ*_2_ in the two stages and the corresponding $\mathcal {R}_{0}$. It is related to the diagnosis and treatment measures nowadays. Although the full coverage of DOTS strategy strengthens the isolation and treatment of the infectious individuals, the treatment rate for latent individuals still stays in a relatively low level. So in our previous study, we fix the recovery rate of exposed (*γ*_1_) as the same value through the two stages, and for a further control of TB, there is still a big space for improvement. Then we consider both *γ*_1_ and *γ*_2_ as control functions in the following strategy. An optimal control model for TB is formulated by extending the system above to include control functions. For continuous system, Pontryagin’s maximum principle is an important tool to solve optimal control problems. The objective function usually reflects the bioscience goal, and it has been used to describe optimal therapy and treatment strategy [35, 36]. In our model, the main aim is to minimize the total number of infected individuals over a finite time interval with the lowest cost possible.

The dynamical control system is

8 $$ \left\{ {\begin{aligned} \frac{dS}{dt}&=\Lambda(1-p)-G(\beta) SI+kV-dS, \\ \frac{dV}{dt}&=\Lambda p-kV-dV, \\ \frac{dE}{dt}&=G(\beta) SI-u_{1}(t)E-\varepsilon E-dE, \\ \frac{dI}{dt}&=\varepsilon E-dI-\sigma I-u_{2}(t)I, \\ \frac{dR}{dt}&=u_{1}(t)E+u_{2}(t)I-dR. \end{aligned}} \right.  $$

It is necessary to do early intervention on the exposed population. In system (8), two control functions *u*_1_(*t*) and *u*_2_(*t*), represent the treatment rates for latent class and infectious class, respectively. We consider state system (8) of ODEs in $\mathbb {R}^{5}$ with the set of admissible control functions given by

$${}{\begin{aligned} \Omega_{1}&=\left\{(u_{1}(t), u_{2}(t))\in L^{1}[0, t_{f}]\times L^{1}[0, t_{f}]| a_{1}\leq u_{1}(t)\right.\\&\left.\leq b_{1}, a_{2}\leq u_{2}(t)\leq b_{2}\right\}, \end{aligned}} $$

and *a*_*i*_,*b*_*i*_(*i*=1,2) are fixed positive constants.

The objective functional is defined as

9 $$ {\begin{aligned} J(u_{1}(t), u_{2}(t))&=\int^{{t_{f}}}_{0}[A_{1}E(t)+A_{2}I(t)+\frac{{B_{1}}}{2}u_{1}^{2}(t)\\ &+\frac{{B_{2}}}{2}u_{2}^{2}(t)]dt. \end{aligned}}  $$

Our target is to minimize the infected individuals (including latent and infectious individuals) as well as the costs required to control TB by treating latent and infectious individuals. The constants *A*_*i*_(*i*=1,2) are the weight constants for class *E* and *I* respectively, *B*_*i*_(*i*=1,2) are the measures of the relevant cost of the interventions associated with *u*_1_(*t*) and *u*_2_(*t*).

An optimal control $(u^{*}_{1}(t), u^{*}_{2}(t))$ satisfies

10 $$ J(u^{*}_{1}(t), u^{*}_{2}(t))=\min\limits_{\Omega_{1}} J(u_{1}(t), u_{2}(t)).  $$

The Hamiltonian *H* associated with (8), (9) and (10) is given by

11 $$ \begin{aligned} H=&A_{1}E(t)+A_{2}I(t)+\frac{{B_{1}}}{2}u_{1}^{2}(t)+\frac{{B_{2}}}{2}u_{2}^{2}(t)\\ &+\lambda_{1}[\Lambda(1-p)-G(\beta) SI+kV-dS]\\ &+\lambda_{2}[\Lambda p-kV-dV]\\ &+\lambda_{3}[G(\beta) SI- u_{1}(t)E-\varepsilon E-dE]\\ &+\lambda_{4}[\varepsilon E-dI-\sigma I-u_{2}(t)I]\\ &+\lambda_{5}[u_{1}(t)E+u_{2}(t)I-dR]. \end{aligned}  $$

where *λ*_*i*_(*i*=1,⋯,5) are adjoint variables, which can be determined by solving the following system of differential equations:

12 $$ {\begin{aligned} \frac{{d\lambda_{1}}}{{dt}}&=-\frac{{\partial H}}{{\partial S}}, \frac{{d\lambda_{2}}}{{dt}}=-\frac{{\partial H}}{{\partial V}}, \frac{{d\lambda_{3}}}{{dt}}=-\frac{{\partial H}}{{\partial E}}, \frac{{d\lambda_{4}}}{{dt}}\\&=-\frac{{\partial H}}{{\partial I}}, \frac{{d\lambda_{5}}}{{dt}}=-\frac{{\partial H}}{{\partial R}} \end{aligned}}  $$

satisfying the transversality condition

13 $$ \lambda_{i}(t_{f})=0,\quad for \quad i=1, \cdots, 5.  $$

By calculating, we have

14 $$ \left\{ \begin{aligned} \frac{{d\lambda_{1}}}{{dt}}&=-\frac{{\partial H}}{{\partial S}}=(\lambda_{1}-\lambda_{3})G(\beta) I+d \lambda_{1}, \\ \frac{{d\lambda_{2}}}{{dt}}&=-\frac{{\partial H}}{{\partial V}}=(\lambda_{2}-\lambda_{1})k+\lambda_{2}d, \\ \frac{{d\lambda_{3}}}{{dt}}&=-\frac{{\partial H}}{{\partial E}}=-A_{1}+(\lambda_{3}-\lambda_{5})u_{1}(t)\\&\quad-(\varepsilon+d)\lambda_{3}-\varepsilon\lambda_{4}, \\ \frac{{d\lambda_{4}}}{{dt}}&=-\frac{{\partial H}}{{\partial I}}=-A_{2}+(\lambda_{1}-\lambda_{3})G(\beta) S\\&\quad+(\lambda_{4}-\lambda_{5})u_{2}(t)+(d+\sigma)\lambda_{4}, \\ \frac{{d\lambda_{5}}}{{dt}}&=-\frac{{\partial H}}{{\partial R}}=d\lambda_{5}. \end{aligned} \right.  $$

And the characterized control set of $(u_{1}^{*}, u_{2}^{*})$ is

15 $$ \left\{ \begin{aligned} u_{1}^{*}(t)&=\max\left\{a_{1}, \min\left\{b_{1}, \frac{(\lambda_{3}-\lambda_{5})E}{B_{1}}\right\}\right\}, \\ u_{2}^{*}(t)&=\max\left\{a_{2}, \min\left\{b_{2}, \frac{(\lambda_{4}-\lambda_{5})I}{B_{2}}\right\}\right\}, \end{aligned} \right.  $$

which is determined by

16 $$ \left\{ \begin{aligned} \frac{\partial H}{\partial u_{1}}&=B_{1}u_{1}(t)-\lambda_{3}E+\lambda_{5}E=0, \\ \frac{\partial H}{\partial u_{2}}&=B_{2}u_{2}(t)-\lambda_{4}I+\lambda_{5}I=0. \end{aligned} \right.  $$

### Numerical simulations

In the foundation of the analysis and research above, we study the optimal control problem for TB. In this section, we perform numerical simulations of the optimal system.

To obtain the optimal solutions, we use an iterative method with the help of Runge-Kutta fourth order procedure. The detail algorithm is as follows:

Step 1: We solve system (8) with an initial guess for the control (*u*_1_(*t*),*u*_2_(*t*)) over the time interval [0,*t*_*f*_] using a forward fourth order Runge-Kutta scheme.

Step 2: With the application of the current iteration solution of the state variables (8) and the transversality conditions *λ*_*i*_(*t*_*f*_)=0,*i*=1,⋯,5, the backward Runge-Kutta fourth order procedure is used to solve adjoint system (14) in the same time interval. The controls are updated by combining the state and adjoint values.

Step 3: Then repeat the iterative process. This situation continues until the values of the unknowns at the previous iteration are very close to the ones at the present iteration.

The time interval for which the optimal control is taken as 20 years here. Based on the goodness of fit for the second stage in “Results” section, we forecast the value of *S*,*V*,*E*,*I* and *R* in 2020 and take them as the initial values of our optimal control which are given by *S*(0)=965740160,*V*(0)=36381737,*E*(0)=115575,*I*(0)=1575311 and *R*(0)=289842548 respectively. Here, we suppose that the vaccine has developed, the protection period of the vaccine is taken as 20 years and the fraction of vaccination is raised to 90%. Other parameters follow the second stage in Table 1. For the latent population, because of its asymptomatic, the diagnose of the latent individuals is harder than the infectious individuals and the cost of treatment in latent population is much lower than the infectious population. To measure the difference between latent and infectious population, the weights in the objective function are *A*_1_=1,*A*_2_=10^3^,*B*_1_=10^3^ and *B*_2_=10^6^ [16]. The bounds on the control are based on the effectiveness of the intervention strategy. It is reasonable to assume that the upper bound for *u*_1_(*t*) is 1 and lower bound is 0; the upper bound for *u*_2_(*t*) is 0.75 and lower bound is 0.

**Fig. 4:**
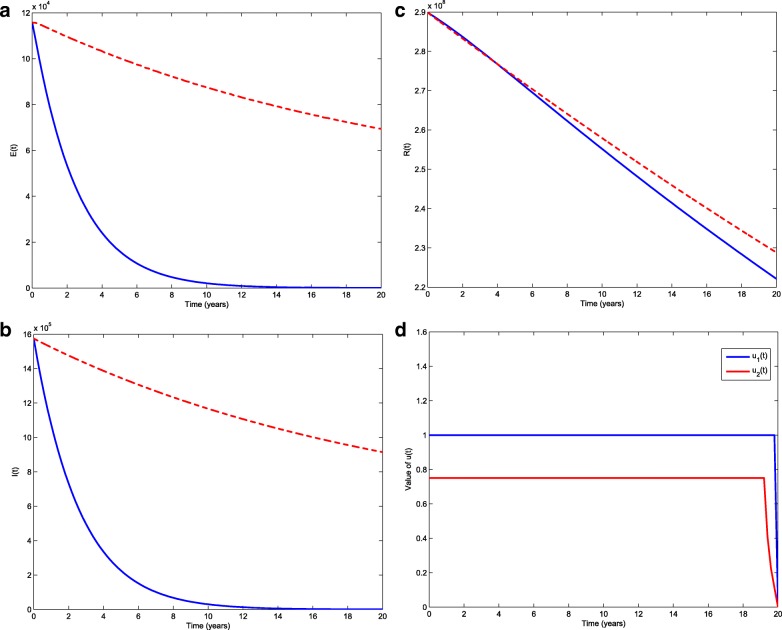
Comparison between the current TB control strategy and optimal control strategy

We compare our optimal control strategy with the results of system (1) under current control strategy which is the situation of the second stage for the following 20 years. In Fig. 4, the population with optimal control actions are shown in blue solid lines and the red dashed lines represent the population under current control strategy. From Fig. 4 we can clearly see that the optimal control strategy gives a better result, that is in the case of optimal control, the decrease is more obvious in the exposed and infectious individuals than in the case of current control strategy.

## Conclusions and discussion

In this paper, we propose mathematical models and use numerical simulations to describe the transmission of TB in China. The aim of system (1) is to reconstruct the past two decades epidemic situation of TB and to forecast the further spread of TB. A compartmental nonlinear deterministic epidemic model is formulated. Theoretic analysis for its global dynamical behaviors is given. $\mathcal {R}_{0}< 1$ ensures the global stability of the infection-free equilibrium. We examine the model with the reported data from 1998 to 2017 in China. The new reported cases of TB change dramatically around the year of 2004 due to the universal coverage of DOTS strategy, so we study the control of TB in China from two different stages, i.e., before and after the full coverage of DOTS strategy. The diagnosis and treatment level are the most important considered factors in our two stage-study. The basic reproduction number for each stage is 1.7885 and 1.0741 respectively. Although the DOTS strategy plays an important role to eliminate TB in China, which is mainly reflected in the basic reproduction number of the two stages, the disease still exists.

Because vaccines and treatment measures are developing fast nowadays, we study further control for TB from the two sides. $\mathcal {R}_{0}$ is reduced by improving the fraction of vaccination and increasing the protection period of vaccine (see Fig. 3). On the other hand, to improve the treatment for early exposed and infected population is also explored. An optimal control problem to minimize the total number of infected individuals with the lowest cost is proposed and analyzed by Pontryagin’s maximum principle. Numerical simulations of the optimal control system provide valuable results to illustrate the theoretical analysis. The optimal strategy is compared with the current strategy in Fig. 4. The discussion of further control for TB indicates that vaccination and treatment strategies are effective in reducing the transmission of TB. Our study provides theoretical support for making further control of TB and help the design of optimal control strategy in practice.

## Appendix

1. Proof of Theorem 1

### *Proof*

The Jacobian matrix of system (1) at *E*_0_ takes the form of

$${\begin{aligned} J_{{E_{0}}}\!\,=\, \left(\begin{array}{ccccc} -d&k&0&-\frac{{G(\beta)\Lambda(k+d-pd)}}{{d(k+d)}}&0\\ 0&-(k+d)&0&0&0\\ 0&0&-(\varepsilon+d+\gamma_{1})&\frac{{G(\beta)\Lambda(k+d-pd)}}{{d(k+d)}}&0\\ 0&0&\varepsilon&-(d+\sigma+\gamma_{2})&0\\ 0&0&\gamma_{1}&\gamma_{2}&-d \end{array} \right). \end{aligned}} $$

It can be calculated that the eigenvalues for the matrix $J_{{E_{0}}}$ are

$$ {\begin{aligned} &\lambda_{1}=-d<0, \\ &\lambda_{2}=-(k+d)<0, \\ &\lambda_{3}=\frac{-(2d+\sigma+\gamma_{2}+\gamma_{1}+\varepsilon)+ \sqrt{(2d+\sigma+\gamma_{2}+\gamma_{1}+\varepsilon)^{2}-4(d+\sigma+ \gamma_{2})(\gamma_{1}+\varepsilon+d)(1-\mathcal{R}_{0})}}{2}, \\ &\lambda_{4}=\frac{{-(2d+\sigma+\gamma_{2}+\gamma_{1}+\varepsilon) -\sqrt{{(2d+\sigma+\gamma_{2}+\gamma_{1}+\varepsilon)^{2}-4(d+\sigma +\gamma_{2})(\gamma_{1}+\varepsilon+d)(1-\mathcal{R}_{0})}}}}{2}, \\ &\lambda_{5}=-d<0. \end{aligned}}  $$

If $\mathcal {R}_{0}<1$, then the real part of *λ*_3_ and *λ*_4_ are negative. Hence the infection-free equilibrium *E*_0_ is locally asymptotically stable. Obviously, if $\mathcal {R}_{0}>1$, then the infection-free equilibrium *E*_0_ is unstable. The proof is completed. □

2. Proof of Theorem 2

### *Proof*

Denote *M*=*S*+*E*+*I*+*R*, it follows that

$$ \begin{aligned} \frac{{dM}}{{dt}}&=\frac{{dS}}{{dt}}+\frac{{dE}}{{dt}}+\frac{{dI}}{{dt}}+\frac{{dR}}{{dt}}\\&=\Lambda(1-p)+kV-dS-dE-dI-\sigma I-dR\\ &=\Lambda(1-p)+k(N-M)-dM-\sigma I\\ &\leq\Lambda(1-p)+kN-(k+d)M\\ &\leq\Lambda(1-p)+\frac{{k\Lambda}}{d}-(k+d)M. \end{aligned}  $$

Then

$$ \lim\limits_{{t\rightarrow+\infty}}\sup M=\frac{{\Lambda(k+d-pd)}}{{d(k+d)}}.  $$

The *S*,*E*,*I* and *R* are all non-negative, $S\leq \frac {{\Lambda (k+d-pd)}}{{d(k+d)}}$.

Define the Lyapunov function

$$ L=\varepsilon E+(\gamma_{1}+\varepsilon+d)I.  $$

Then the derivative of *L* with respect to *t* along the solutions of system (1) is given by

17 $$ {\begin{aligned} \frac{{dL}}{{dt}}&=\varepsilon\frac{{dE}}{{dt}}+(\gamma_{1}+\varepsilon+d)\frac{{dI}}{{dt}}\\ &=(\gamma_{1}+\varepsilon+d)(d+\sigma+\gamma_{2})\left(\mathcal{R}_{0}\frac{{d(k+d)}}{{\Lambda(k+d-pd)}}S-1\right)I. \end{aligned}}  $$

If $\mathcal {R}_{0}< 1$ holds, *dL*/*dt*<0. Therefore, the Lyapunov-Lasalle theorem guarantees the global stability of the infection-free equilibrium *E*_0_. The proof is completed. □

## Data Availability

The data in the manuscript is published by National Bureau of Statistics of China, as given in the References.

## References

[1] World Health Report 2001: Global Tuberculosis Control. http://apps.who.int/iris/bitstream/10665/63835/4/WHO_CDS_TB_2001.287.pdf.

[2] Global Tuberculosis Report 2018. https://apps.who.int/iris/bitstream/handle/10665/274453/9789241565646-eng.pdf?ua=1.

[3] Xu B, Hu Y, Zhao Q, Wang W, Jiang W, Zhao G (2015). Molecular epidemiology of tb - its impact on multidrug-resistant tuberculosis control in china. Int J Mycobact.

[4] Pinto E, Nepomuceno E, Campanharo A. Influence of contact network topology on the spread of tuberculosis, vol. 1068: Springer; 2019, pp. 81–88. 10.1007/978-3-030-36636-0_6.

[5] Aparicio J, Castillo-Chavez C (2009). Mathematical modelling of tuberculosis epidemics. Math Biosci Eng.

[6] Song B, Castillo-Chavez C, Aparicio J (2002). Tuberculosis models with fast and slow dynamics: The role of close and casual contacts. Math Biosci.

[7] Blower S, Chou T (2004). Modeling the emergence of the ‘hot zones’: Tuberculosis and the amplification dynamics of drug resistance. Nat Med.

[8] Khajanchi S, Das D, Kar T (2018). Dynamics of tuberculosis transmission with exogenous reinfections and endogenous reactivation. Physica A.

[9] Das D, Khajanchi S, Kar T (2020). Transmission dynamics of tuberculosis with multiple re-infections. Chaos Soliton Fract.

[10] Sharomi O, Podder C, Gumel A, Song B (2008). Mathematical analysis of the transmission dynamics of hiv/tb coinfection in the presence of treatment. Math Biosci Eng.

[11] Zhou Y, Khan K, Feng Z, Wu J (2008). Projection of tuberculosis incidence with increasing immigration trends. J Theor Biol.

[12] Liu L, Zhao X, Zhou Y (2010). A tuberculosis model with seasonality. Bull Math Biol.

[13] Blower S, Small P, Hopewell P (1996). Control strategies for tuberculosis epidemic: New models for old problems. Science.

[14] Mondal P, Kar T (2017). Optimal treatment control and bifurcation analysis of a tuberculosis model with effect of multiple re-infections. Int J Dynam Control.

[15] Castillo-Chavez C, Feng Z (1998). Global stability of an age-structure model for tb and its applications to optimal vaccination strategies. Math Biosci.

[16] Yang Y, Tang S, Ren X, Zhao H, Guo C (2016). Global stability and optimal control for a tuberculosis model with vaccination and treatment. Discret Cont. Dyn-B..

[17] Nepomuceno E, Takahashi R, Aguirre L (2018). Reducing vaccination level to eradicate a disease by means of a mixed control with isolation. Biomed Signal Process.

[18] Liu S, Yang X, Bi Y, Li Y (2019). Dynamic behavior and optimal scheduling for mixed vaccination strategy with temporary immunity. Discrete Cont Dyn-B.

[19] Das D, Khajanchi S, Kar T (2020). The impact of the media awareness and optimal strategy on the prevalence of tuberculosis. Appl Math Comput.

[20] Khajanchi S (2019). Stability analysis of a mathematical model for glioma-immune interaction under optimal therapy. Int J Nonlin Sci Num.

[21] Nepomuceno E, Barbosa A, Silva M, Perc M (2018). Individual-based modelling and control of bovine brucellosis. Roy Soc Open Sci.

[22] Moualeu D, Weiser M, Ehrig R, Deuflhard P (2015). Optimal control for a tuberculosis model with undetected cases in cameroon. Commun Nonlinear Sci Numer Simul.

[23] Rodrigues P, Silva C, Torres D (2014). Cost-effectiveness analysis of optimal control measures for tuberculosis. Bull Math Biol.

[24] Driessche P, Watmough J (2002). Reproduction numbers and sub-threshold endemic equilibria for compartmental models of disease transmission. Math Biosci.

[25] National Bureau of Statistics of China, Statistical Data of Tuberculosis 1998-2017. http://data.stats.gov.cn/easyquery.htm?cn=C01&zb=A0O0F01.

[26] National Bureau of Statistics of China, China Statistical Yearbook 2000, Birth Rate, Death Rate and Natural Growth Rate of Population. http://www.stats.gov.cn/tjsj/ndsj/zgnj/2000/D02c.htm.

[27] National Bureau of Statistics of China, China Statistical Yearbook 2014, Birth Rate, Death Rate and Natural Growth Rate of Population. http://www.stats.gov.cn/tjsj/ndsj/2014/indexch.htm.

[28] Ziv E, Daley C, Blower S (2001). Early therapy for latent tuberculosis infection. Am J Epidemiol.

[29] Li J (2013). The spread and prevention of tuberculosis. Chin Rem Clin.

[30] Chang H (2013). Quality monitoring and effect evaluation of bcg vaccination in neonatus. Occup Health.

[31] Xiong C, Liang X, Wang H (2009). A systematic review on the protective efficacy of bcg against children tuberculosis meningitis and millet tuberculosis. Chin J Vacc Immun.

[32] Cao H, Zhou Y (2012). The discrete age-structured seit model with application to tuberculosis transmission in china. Math Comput Model.

[33] Lopes J, Rodrigues P, Pinho S, Andrade R, Duarte R, Gomes M (2014). Interpreting measures of tuberculosis transmission: A case study on the portuguese population. BMC Infect Dis.

[34] Liu S, Li Y, Bi Y, Huang Q (2017). Mixed vaccination strategy for the control of tuberculosis: a case study in china. Math Biosci Eng.

[35] Khajanchi S, Ghosh D (2015). The combined effects of optimal control in cancer remission. Appl Math Comput.

[36] Khajanchi S, Banerjee S (2019). A strategy of optimal efficacy of t11 target structure in the treatment of brain tumor. J Biol Syst.

